# Experiences with Oral Pre-Exposure Prophylaxis (PrEP) Service Delivery and Use Among Adolescent Girls and Young Women (AGYW) in Routine Primary Care Settings, South Africa

**DOI:** 10.1007/s10461-024-04596-5

**Published:** 2025-01-09

**Authors:** Catherine E. Martin, Lorrein S. Muhwava, Siphokazi Dada, Fiona Scorgie, Saiqa Mullick

**Affiliations:** Wits RHI, University of Witwatersrand, Johannesburg, South Africa

**Keywords:** HIV prevention, Pre-exposure Prophylaxis (PrEP), Adolescent girls and young women (AGYW), Health service delivery, PrEP use, Socio-Ecological Model (SEM), South Africa

## Abstract

In South Africa, oral PrEP was included in national guidelines as part of a combination HIV prevention package for adolescent girls and young women (AGYW) in 2017. Understanding their experiences of accessing and using PrEP is necessary to evaluate and improve PrEP service delivery approaches. This descriptive study explored AGYW’s knowledge and understanding of PrEP, their experiences and influences on PrEP access and use in real world settings in South Africa. We conducted 44 in-depth interviews with female PrEP users (15-24 years) enrolled in Project PrEP. Interviews were audio recorded and transcribed for analysis using thematic analysis procedures. Participants reported positive experiences and overall satisfaction with accessing health services (i.e., youth-friendly clinic spaces, friendly and non-judgemental staff, privacy during consultations). Distance from the clinic, long queues, negative provider attitudes, and occasional stock-outs were key health service barriers to accessing PrEP. Individual motivating factors to continued PrEP use included creating daily pilltaking routines and the goal to remain HIV negative, while barriers included pill fatigue, frequent clinic visits, and side-effects. Positive relationships with partners and family facilitated disclosure of PrEP use, while stigma was identified as a community barrier to accessing PrEP services. Our study highlights AGYWs' experiences with PrEP access and use in a real-world setting. Facilitators and barriers identified in this study can be leveraged to strengthen efforts to support young women and ensure effective PrEP use. The findings also contribute to the development of appropriate service delivery.

## Background

Adolescent girls and young women (AGYW) between the ages of 15 and 24 years are disproportionately affected by HIV. In 2023 they accounted for 44% of new HIV infections globally, with the estimated number of new infections four times higher than the 2025 target [1]. In South Africa, the prevalence of HIV among AGYW is estimated to be twice as high as adolescent boys and young men [2]. Strengthening HIV prevention in this population is imperative. Pre-exposure prophylaxis (PrEP) has become the standard for HIV prevention globally and is recommended by the World Health Organisation (WHO) as part of a combination HIV prevention package for at risk groups, including AGYW [3]. In 2017, oral PrEP was included in South Africa’s national HIV prevention guidelines for AGYW and has subsequently been rolled out in 95% of the country’s primary healthcare facilities to over 1,6 million individuals [4]. Despite its effectiveness in reducing HIV transmission, many challenges with PrEP access, uptake, and effective use remain [5]. Early discontinuation of oral PrEP among AGYW has been reported in PrEP implementation programmes [6–10], highlighting a need to better understand the facilitators and barriers to effective PrEP use in real-world settings. In particular, there is a need to explore the service delivery and health systems factors which may influence PrEP uptake and use within integrated sexual and reproductive health (SRH) service delivery.

Qualitative studies on health providers’ knowledge, attitudes, and experiences with providing PrEP to the general population [11] and to young women [12–14] highlight important health system factors that impact provision of PrEP such as limited resources, provider reservations about PrEP, and judgemental attitudes towards young women’s sexual behaviour. Whilst client-related challenges such as transport costs, pill fatigue, and side effects have been well documented [7, 15, 16], there are fewer data on the broader facilitators and barriers to PrEP access and use in real-world settings. Studies among young women have highlighted the importance of awareness of vulnerability, support from families, partners and friends as well as service convenience in ongoing PrEP use, noting barriers to service access that may be created by negative healthcare provider attitudes, lack of confidentiality and stigma [7, 8, 10, 15–18].

The introduction of long-acting PrEP methods, such as the dapivirine vaginal ring, injectable cabotegravir and injectable lenacapavir, could offer additional HIV prevention options for women and potentially eliminate some of the perceived and actual barriers associated with oral PrEP use, such as poor adherence, pill fatigue, and stigma [5]. However, delivery of effective, integrated and acceptable HIV prevention services will remain critical to PrEP access and use regardless of the PrEP method. In order to strengthen the integration of PrEP methods within existing services, we aimed to broaden our understanding of young women’s experiences of accessing and using oral PrEP in routine service delivery settings, as well to build a comprehensive understanding of barriers and facilitators to PrEP access and use within the context of routine delivery of integrated HIV prevention services. In addition, in order to properly contextualise and strengthen the implementation of service delivery models that are acceptable to girls and young women, that address known barriers, and that build on the strengths of existing services, we aimed to understand the factors that influence young women’s decision-making and use of PrEP when integrated within routine SRH services.

## Theoretical Framework

It is well understood that vulnerability to HIV is influenced by multiple levels that go beyond individual knowledge and attitudes, and that a more holistic and structural approach to HIV prevention is required [19]. In order to more comprehensively understand the broader influences on women’s decision making with regards to PrEP access and use, we applied the Centers for Disease Control and Prevention’s Socio-Ecological Model (SEM) [20]. This model provides a useful framework for understanding the multi-level factors that influence health behaviour and may be useful in guiding multi-level interventions [19, 21]. It has been used in other HIV prevention studies to contextualise facilitators and barriers to uptake of prevention methods, use and service delivery [15, 22]. The SEM is made up of four constructs, each representing a level of influence namely: (i) individual factors such as knowledge, attitudes, and personal beliefs (ii) interpersonal/relationship, such as the influence of partners, family and peers; (iii) health system, such as service delivery models and provider attitudes and (iv) community/societal factors such as social norms and community beliefs or myths [15, 22].

The objective of this descriptive study was to explore the factors influencing oral PrEP access and use among AGYW accessing oral PrEP in routine, primary care settings during the introduction of oral PrEP within national health services in South Africa.

## Methods

### Study Design

This descriptive qualitative study made use of in-depth interviews to explore individual perceptions and obtain in-depth insights into AGYW’s experiences. The Consolidated Criteria for Reporting Qualitative studies (COREQ) checklist was followed to ensure rigour in the study [23], and we applied the Socio-Ecological Model to frame the data analysis and insights generated.

### Study Setting

The study is embedded in Wits RHI’s Unitaid funded ‘Project PrEP’, an implementation science study which aimed to generate evidence to inform the introduction and integration of PrEP within combination HIV prevention and SRH services, targeting AGYW, in South Africa [24]. The project was implemented in four geographical clusters in South Africa; one peri-urban and one rural cluster in the Eastern Cape, one urban cluster in Kwa-Zulu Natal and one peri-urban cluster in Gauteng. Study sites consist of eight fixed Department of Health primary care facilities, and four linked, roving mobile clinics. Sites offer integrated HIV prevention and SRH services, including HIV testing, contraception, sexually transmitted infection (STI) management, and PrEP, with linkage to other prevention or treatment services as required. Sites collaborate with local community-based organizations to generate demand for SRH and HIV prevention services, in addition to the use of national online digital platforms targeting young people [25, 26].

### Study Participants and Data Collection

Between December 2018 and December 2021, the project initiated 21 983 clients on PrEP, of whom 14 637 were AGYW, and 1170 AGYW enrolled in a nested longitudinal cohort. A subset of this cohort was purposively selected and invited to participate in an in-depth interview (IDI). Participants were selected based on the following inclusion criteria: female (15–24 years) who had initiated PrEP at one of the study facilities; living or working in the study catchment areas or communities; and able and willing to provide written informed consent (and assent for minors) for participation and digital recording of the IDIs. Data were collected between February 2020 and November 2021, with a total of 44 participants from across all four geographical clusters. Eligible participants enrolled in the cohort study were approached telephonically by study staff and invited to participate in an IDI. The IDIs were conducted by trained female researchers, in either English or the participant’s home language (e.g., isiZulu, isiXhosa, and Setswana) at an agreed date and time at the study sites. All participants were black African and were matched with a researcher fluent in their preferred language to ensure that language would not be a barrier to understanding during the IDI. Owing to the global COVID-19 pandemic and lockdown restrictions in South Africa during the data collection period, some interviews were conducted telephonically to accommodate participants who were unable to travel to the study site.

An interview guide, developed by the researchers, was used to direct the discussion, with probes adapted as data collection proceeded. The guide included questions about participants’ knowledge and understanding of PrEP, their decision to use PrEP, their challenges or successes with PrEP use, and their experiences accessing PrEP services, among others. IDIs were conducted by a researcher or fieldworker in a private, quiet space, away from clinic activities to avoid disruptions, and lasted between 15 and 120 min, depending on the participant’s responsiveness. All interviews were recorded with the participant’s consent and professionally transcribed. Interviews that were conducted in a language other than English were transcribed by members of the project team who were fluent in the source language, and thereafter translated into English for data analysis. Audio files and transcripts were stored on a secure server with restricted access. Data collection was conducted until a minimum sample per geographical area had been recruited.

### Data Analysis

All transcripts were reviewed for accuracy by two qualitative researchers (LSM and SD), prior to data analysis. The data analysis process was inductive and followed Braun and Clarke’s six phases of thematic analysis procedures [27], which involved (i) familiarisation with the data, (ii) generating initial codes, (iii) searching for themes, (iv) reviewing the themes, (v) defining the themes and finally, (vi) writing up the findings. NVivo 12 (Version 1.6.1) was used for data management and coding of the transcripts. The analysis team consisted of three qualitative researchers (LSM, SD and FS) and a medically trained, HIV technical specialist (CM). The coding framework was developed iteratively. Two researchers (LSM and SD) initially coded two transcripts independently using open coding to familiarise with the data and develop a draft coding framework. A third researcher (FS) reviewed the initial codebooks, which were then revised based on feedback from weekly meetings and discussions with the HIV technical specialist (CM). To ensure consistency across the data, the two coders (LSM and SD) met regularly to discuss their draft coding frameworks and emerging patterns across the data before merging the coding frameworks. FS and CM reviewed the merged coding framework and once it was finalised, LSM and SD independently coded the remaining transcripts in NVivo 12. Once coding was completed, LSM and SD prepared memos highlighting and summarizing emerging themes across the data. The analysis team then met to discuss the findings and finalise the key themes for write-up of the manuscript.

## Results

The demographic and behavioural characteristics of study participants are presented in Table 1. The median age across the sample was 20 years (Inter Quartile Range (IQR): 19;22), and the majority of participants were currently in school or tertiary education and unemployed. One third of participants lived with both parents, and another third with their mother only. 7% of participants reported early sexual debut (at age 14 years or younger). The majority were currently in relationships, and with partners within 5 years of their own age. Of those in relationships, 11.9% had more than one partner. 9% had transactional sex (sex in exchange for money, goods, or other benefits), while one fifth of the sample (18%) had had sex under the influence of drugs or alcohol and used condoms consistently (18%) in the last 3 months.

**Table 1:** Demographic and behavioural characteristics of the study participants

	Site									
	Mthatha (*n* = 8, 18%) *n* (%)		Gqeberha (*n* = 11, 25%) *n* (%)		Tshwane (*n* = 10, 23%) *n* (%)		KZN (*n* = 15, 34%) *n* (%)		Total (*N* = 44, 100%) *n* (%)	
**Age group**										
15–17 years	2	25.0%	2	18.2%	2	20.0%	0	0.0%	6	13.6%
18–20 years	3	37.5%	4	36.4%	4	40.0%	8	53.3%	19	43.2%
21–24 years	3	37.5%	5	45.5%	4	40.0%	7	46.7%	19	43.2%
**Sexual orientation**										
Heterosexual	8	100.0%	9	81.8%	10	100.0%	15	100.0%	42	95.5%
Non-heterosexual	0	0.0%	2	18.2%	0	0.0%	0	0.0%	2	4.5%
**Currently in school or tertiary? education**	5	62.5%	7	63.6%	6	60.0%	12	80.0%	30	68.2%
**Employment status**										
Employed	1	12.5%	1	9.1%	1	10.0%	4	28.6%	7	16.3%
Unemployed	7	87.5%	10	90.9%	9	90.0%	10	71.4%	36	83.7%
**Household primary adult caregiver**										
Mother only	2	25.0%	2	18.2%	6	60.0%	3	20.0%	13	29.5%
Father only	2	25.0%	1	9.1%	0	0.0%	0	0.0%	3	6.8%
Mother and Father	1	12.5%	4	36.4%	4	40.0%	5	33.3%	14	31.8%
Adult relation	1	12.5%	3	27.3%	0	0.0%	0	0.0%	4	9.1%
Neither parent nor adult relation	2	25.0%	1	9.1%	0	0.0%	7	46.7%	10	22.7%
**Early sexual debut**	1	12.5%	0	0.0%	2	20.0%	0	0.0%	3	6.8%
**Current relationship status**										
In a relationship	8	100.0%	9	81.8%	9	90.0%	13	86.7%	39	88.6%
Single /no partner	0	0.0%	2	18.2%	1	10.0%	2	13.3%	5	11.4%
**Partner’s age difference**										
≤ 5 years	4	50.0%	9	81.8%	7	70.0%	13	86.7%	33	75.0%
> 5 years	4	50.0%	1	9.1%	2	20.0%	0	0.0%	7	15.9%
Unknown	0	0.0%	1	9.1%	1	10.0%	2	13.3%	4	9.1%
**Additional partners**										
Yes	2	25.00%	1	10.0%	1	10.0%	1	7.14%	5	11.9%
Transactional sex*	1	12.5%	2	18.2%	1	10.0%	0	0.0%	4	9.1%
Sex under the influence of alcohol or drugs*	2	25.0%	2	18.2%	1	10.0%	3	20.0%	8	18.2%
Consistent condom use*	1	12.5%	3	27.3%	1	10.0%	3	20.0%	8	18.2%
*In the last 3 months										

**Fig. 1:**
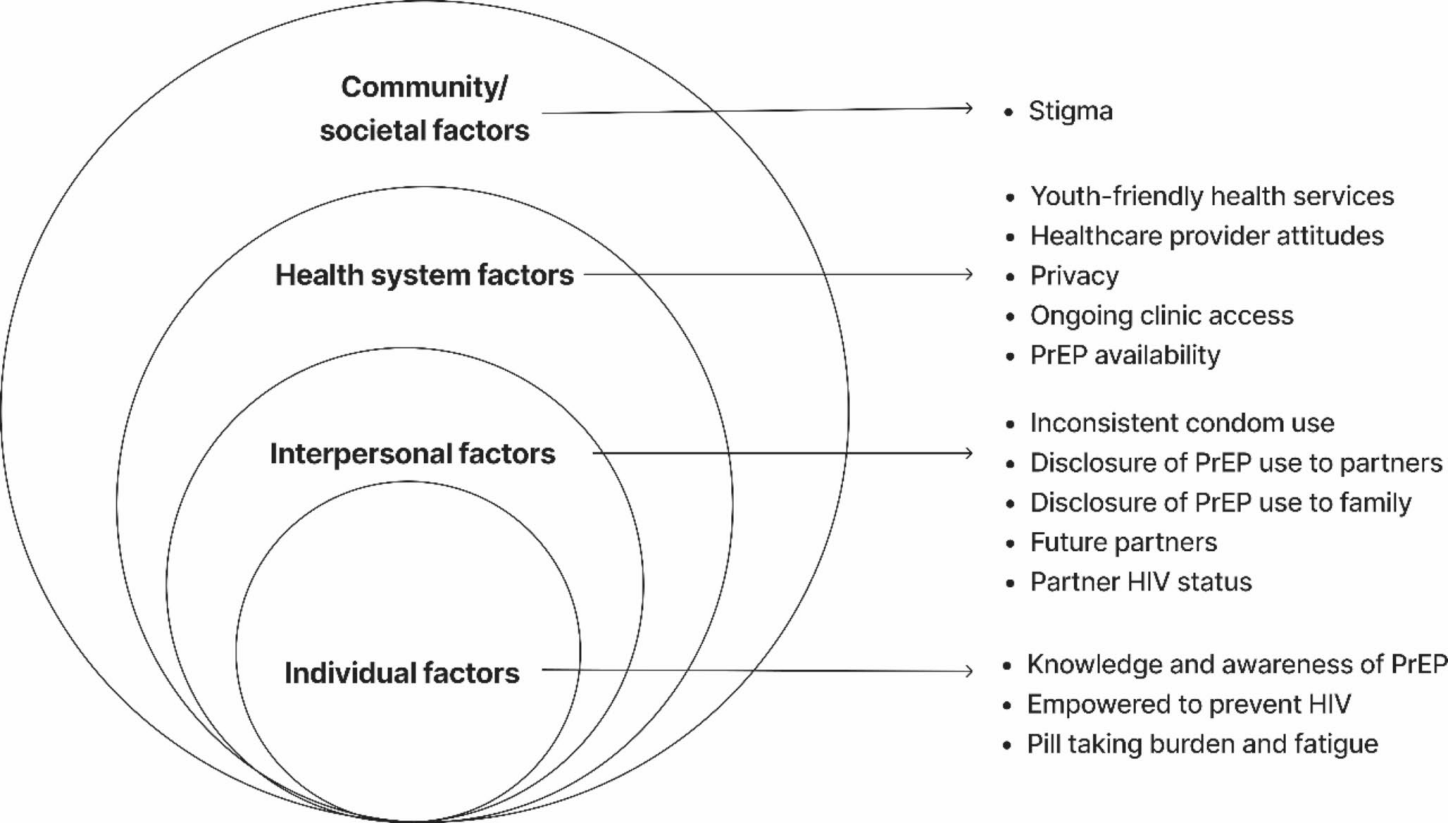
Socio-ecological model of factors influencing PrEP access and use among AGYW in South Africa

Findings below are presented according to the SEM constructs, documenting the (i) individual, (ii) interpersonal, (iii) health system and (iv) community/societal factors influencing young women’s access and use of PrEP within integrated, primary care services (Fig. 1).

### Individual Factors

#### Knowledge and Awareness of PrEP

Most participants demonstrated a good current understanding of PrEP as an HIV prevention method or “*prevention pill*”. Participants attributed their knowledge of PrEP and decision to initiate PrEP to the provision of counselling and educational resources by Project PrEP in community clinics, mobile clinics and schools. One participant describes how the information received about PrEP when she visited the clinic supported her decision to initiate PrEP.

*I was also one of those people who were saying that when you take PrEP you are infecting yourself with HIV. Then I went to the clinic to get tested for HIV and I was asked if I know anything about PrEP and I told them I don’t. After my results came back*,* the nurse told me more about PrEP and said if I want to take it*,* I can take it*,* it’s available. Then I decided to take it.****(20 years old***,*** 4 months on PrEP)***.

Another describes how hearing about something to prevent HIV was novel and peaked her interest.

*I was surprised because there’s been no cure for HIV for a very long time*,* but to finally find that there is something to help prevent*,* I was surprised and interested at the same time.****(19 years old***,*** 4 months on PrEP)***.

A few participants mentioned hearing about PrEP through sources outside the clinic, including television, social media, and school-based education, as one participant describes:

*I heard about PrEP on MTV Shuga [a television series promoting HIV prevention and positive sexual health] was playing and there was an episode about people living with HIV and how they contracted it and how they live*,* how society treats them and how you can prevent yourself from getting HIV if you have a partner that you don’t trust or like*,* [if] he forces you to sleep with him without using a condom. So*,* this lady was like*,* “There is something called PrEP’. That was the first time I heard of it. I was like*,* “what is this thing?” And at the time*,* it was only offered in Joburg. Then there are people who came to my school when I was still in high school*,* telling us about PrEP*,* and I was like*,* “I heard about this on TV”. Then they were like*,* “Come to the clinic and try it out”.****(19 years old***,*** 6 months on PrEP)***.

Although most participants felt that the educational materials provided by Project PrEP were sufficiently informative, there were two key suggestions to improve their effectiveness: to include more information to empower young women, and to broaden the focus of the materials to encourage young men to also take PrEP.

#### Desire to be Empowered to Prevent HIV

PrEP uptake and continued use were primarily influenced by a desire to prevent HIV infection and be a part of an HIV-free generation. Many participants personally identified with the project slogan “*We are the generation that will end HIV*” which resonated with their views of taking PrEP as empowering themselves to take control of their health.

Several participants perceived themselves to be at risk for sexual violence and cited a fear of acquiring HIV as a result of rape as a key factor motivating them to take PrEP and stay on it. As two participants explain:

*The fact that I take PrEP every day to limit my chances of contracting the virus and the fact that I could get caught up in unexpected situations like being violated. That is what motivates [me] to keep taking PrEP right through.****(20 years old***,*** 2 years 1 month on PrEP)***.

*There are thugs in the neighbourhood*,* they may break in and rape us*,* that is what I am scared of.****(15 years old***,*** 9 months on PrEP)***.

Participants also described individual strategies to support them to continue taking PrEP such as creating daily pill-taking routines and setting reminders (alarms) on their mobile phones to avoid missing doses.

#### Pill Taking Burden and Fatigue

Although a few participants reported no challenges with PrEP use at all, some participants described challenges with pill fatigue and forgetfulness. The burden of taking a daily pill was reported as a common challenge, with many participants complaining about the size of the pill, which they felt was too big to swallow.

*The thought of taking PrEP is tiring*,* every day is tiring… it is a lot of work*,* and the pill… is also so big…. truly is big.****(20 years old***,*** 9 months on PrEP)***.

Additionally, as one participant explained, pills were viewed as something that sick people use, and not those who are well.

*Taking the pill at the exact time every day. That has been a bit challenging because I’m not a person who gets sick*,* I’m not a person who takes pills.****(19 years old***,*** 6 months on PrEP)***.

*Yeah*,* sometimes I think “Argh*,* why do I have to take it?” But then I have to protect myself*,* so I have to.****(19 years old***,*** 4 months on PrEP)***.

A few participants felt that alternative forms of PrEP such as an injection, would be preferrable to the pills.

*For me*,* the pill is an issue because we had some discussion with my room mates*,* and they understand*,* but we thought it would be better if….PrEP was an injection.****(20 years old***,*** 7 months on PrEP)***.

Common side effects of PrEP such as nausea, vomiting, headache and diarrhoea were noted by many participants. However, most participants reported only experiencing these side effects in the first few days of taking PrEP, which resolved over time.

### Interpersonal Factors

Relationships with partners, family and friends were seen to have an influence on AGYW’s access to and use of PrEP. Positive relationships with partners and family facilitated disclosure of PrEP use and encouraged support for continued use of PrEP. Inconsistent condom use and partners’ HIV status also played a role.

#### Inconsistent Condom Use

Most participants demonstrated a high awareness of personal vulnerability to HIV acquisition. Inconsistent condom use and partner mistrust were reported by AGYW in long-distance relationships as common reasons for initiating PrEP. Although many participants were aware of the importance of condom use as a preventative measure against contracting HIV and STIs, some reported that they had difficulty negotiating condom use with their partners, and this led to inconsistent use. A few participants also reported that they themselves had more than one sexual partner. Here a participant explains her rationale for starting PrEP, noting her vulnerability in not using condoms and having more than one partner.

*My motivation was that I was dating someone else when I started dating my current partner*,* so*,* it was like dating two people at the same time. I was like*,* okay*,* if I get infected with HIV*,* I will not even know who I got it from. I was not using a condom with the other partner*,* and I was using it with this one*,* but I saw how strong we were during the intercourse. Then I decided that I had to protect myself*,* I did not take it [PrEP] the same time*,* I decided after he told me that he is taking ARVs.****(24 years old***,*** 9 months on PrEP)***.

#### Disclosure of PrEP Use to Intimate Partners

Many participants were unsure of or did not know their partner’s HIV status. Some participants reported disclosing their PrEP use to their partners and receiving support and encouragement to continue with taking it,

*Yes*,* he [my partner] does support me. He would ask about my appointment date or ask if I have been to the clinic to take my tablets.****(24 years old***,*** 19 months on PrEP)***.

However, others had no intention to disclose PrEP use to their partners out of concern that they may not approve, or their partners would then discontinue condom use, putting them at risk for STIs.

*[I have not disclosed to] my boyfriend because I am not sure how he will react*,* and I suspect he might not want to use protection with me [although] using PrEP does not mean you must stop using condoms. I am scared to tell him as I want to be careful…. he might want to take advantage that I am taking PrEP. There are other diseases like STIs*,* so I want to be extra careful.****(22 years old***,*** 4 months on PrEP)***.

According to one participant, her use of PrEP was initially perceived by her partner to indicate a lack of trust, although when he became aware she was participating in a study, and explained she was using PrEP for her own health, his views changed and he became supportive. She described how overhearing a study interview on the phone provided an opportunity to discuss her PrEP use.

*After that I kept getting calls*,* doing telephonic interviews*,* that is when he started being supportive. He would listen and like*,* hear my answers*,* like*,* he was supportive. After I told him that “Listen*,* it is not about me trusting you*,* it’s about my health. It is either you are with me or you just…” After that he was okay.****(19 years old***,*** 6 months on PrEP)***.

#### Disclosure of PrEP Use to Family

Most participants had disclosed their PrEP use to their parents or other family members who had responded positively and offered support. One participant described how her family members often reminded her to take her daily pill on time and had set reminders on their phones to ensure that she did not forget to take her PrEP.

Nonetheless, there were participants who preferred not to disclose to their parents out of fear that they would react negatively and discourage them from using PrEP. Many women reported that their parents were unaware that they were accessing SRH services or even that they were sexually active as these were viewed as uncomfortable topics for both parties. One participant acknowledges the fear of being judged by her mother should she have come to know about her PrEP use.

*I will not tell my mom*,* I am scared she might judge me…. I do not talk to my mom about such things.****(20 years old***,*** 9 months on PrEP)***.

#### Future Partners

An important finding was that a few PrEP users in our study were not currently in any relationship and were not sexually active but were motivated to use PrEP to be prepared “*just in case”* they meet a new partner. As one participant highlights, the future is not always predictable, and it was safer to be prepared,

*See*,* it motivates me*,* because as I am single*,* it could happen that I get someone else anytime soon. So*,* I don’t want to stop using it and get a new partner because I don’t know what will happen. I’d rather continue with it*,* for the future.****(17 years old***,*** 11 months on PrEP)***.

#### Partner HIV Status

Although many participants reported not knowing their partner’s HIV status, some were motivated to use PrEP because they had partners who were known to be HIV positive.

*I started using PrEP because my partner told me he’s on ARVs. That was the point where we decided that we are no longer going to use condoms*,* then he told me his status and I said*,* “It’s fine*,* I’m going to use PrEP”.****(24 years old***,*** 9 months on PrEP)***.

*[I started taking PrEP] because of my partner. I am dating a boy who is HIV positive*,* and I am negative. That is why I decided to take PrEP.****(19 years old***,*** < 1 week on PrEP)***.

### Health System-Related Factors

#### Youth-Friendly Health Services

Most participants reported positive experiences and overall satisfaction with accessing health services. They spoke positively of youth-friendly clinics where young people had designated spaces and were prioritised by health providers to receive integrated health services, as illustrated by one participant:

*They treat me well. I get first preference; I go straight to the sister and get help. I have never even been late to school.****(15 years old***,*** 9 months on PrEP)***.

#### Healthcare Provider Attitudes

Participants also appreciated encountering friendly and non-judgemental healthcare providers who provided them with health information, counselling, and clinical services in a respectful manner, at times going above and beyond for their clients.

*Yes*,* I am [satisfied with the services]. They [nurses] are open people*,* we chat and joke. Others sacrifice their lunch hour or work overtime. They knock off at 5 instead of 4*,* just to help people.****(17 years old***,*** 11 months on PrEP)***.

Participants noted how this facilitated the consultation, allowing them to feel comfortable and speak freely, resulting in an overall positive experience.

*They are sweet! Sweet! Sweet! You don’t even feel like you are talking to a stranger*,* you feel like you are talking to someone you know*,* someone you’ve known for a long time*,* so going to the clinic is like going to see your friend.****(19 years old***,*** 8 months on PrEP)***.

*They are really nice; they can talk to people. They are not strict*,* they don’t judge or swear at people*,* no. Yes*,* I can talk to them and it’s nice to talk to them*. ***(18 years old***,*** 10 months on PrEP)***

For some, the practical and emotional support they had received from healthcare providers at the clinic where they had initiated PrEP had influenced their decision to take PrEP and to continue using PrEP as they felt they still had access to the nurses and other healthcare providers if they faced any challenges or had concerns.

*They [nurses] do explain a lot about PrEP. They even say we must not stop taking PrEP because it is protecting us. They say that we must not miss a dose*,* because there are people who get HIV positive while on PrEP because of [missing] the dose. They do explain. They even gave me the PrEP material.****(15 years old***,*** 9 months on PrEP)***.

Where health providers had negative attitudes towards AGYW, this was noted to create a communication barrier and prevented women from openly sharing their challenges or concerns, out of fear of being judged. One participant contrasted how a friendly service facilitated engagement, but being unfriendly and disrespectful created barriers.

*I think if nurses can be open and friendly with us this can help. As people we can be able to feel free to talk to them. But the moment they give me an unfriendly service I will not be able to share my challenges with them. Some nurses are shouting us and other can hear our challenges*,* they just need to be friendly and engage us respectfully. I do not like it…….****(20 years old***,*** 7 months on PrEP)***.

#### Privacy

Privacy during consultations was highlighted as a key factor in facilitating access to PrEP as participants felt they could express themselves freely to healthcare providers, who assured them that their discussions would remain confidential. A small minority reported negative experiences, however. One young women details how she had once overheard healthcare providers discussing other patients,

*Oh*,* sometimes the nurses gossip about us. Uhm*,* when I was there*,* a patient had just left so there were two nurses in the room*,* and I heard them talking about her. I was sad because I thought that maybe sometimes*,* they talk about me.****(16 years old***,*** 10 months on PrEP)***.

Another reports how the limited space in the clinic and use of curtains as a barrier meant that their consultation would inevitably be heard by others.

*Privacy*,* I think*,* is not enough because the other will be pumping blood [i.e. taking blood pressure] that side*,* then when you go to the other side*,* because they have divided with… for instance*,* it looks like a cloth*,* so when you go and test*,* I feel like other people can hear your business. Yah*,* so I feel that the space is too little.****(22 years old***,*** 9 months on PrEP)***.

#### Ongoing Clinic Access

Despite their motivation to take PrEP and having developed strategies to avoid missing doses, some participants reported time periods where they had stopped taking PrEP due to challenges with travelling to the clinic. This was mostly because they lived a long distance from the clinics and transport costs were high.

*[My biggest challenge] is not having access*,* it’s only one*,* not having access to it…the fact that I stay far from the clinic*,* and I have to use public transport when I go there; and I don’t always have transport fare in that week; I’d have it the following week.****(20 years old***,*** 4 months on PrEP)***.

They also bemoaned the frequent clinic visits (every month) and long waiting times at the clinic.

*The dates must be spread apart…when dates are close to each other…. Aaai… it is a problem. They must increase the dosage of medication*,* maybe from the boxes of medication*,* increase dosage of medication…. speed up the process to be shorter to avoid patients waiting for too long.****(22 years old***,*** 4 months on PrEP)***.

In addition, COVID-19 restrictions on movement during the implementation of Project PrEP prevented some participants from accessing health services including PrEP. Many participants were still attending school and would often arrive late at the clinic after the school day had ended, and had to stand in long queues or, in some cases, even miss their scheduled appointment altogether.

#### Availability of PrEP

Another key health service barrier to accessing PrEP was the occasional stock-out of PrEP in some clinics. Some non-project clinics did not offer PrEP and hence when they travelled back home during the school holidays, AGYW would have to stop taking PrEP if they ran out of medication.

### Community/Societal Factors

#### Stigma

Some participants feared that disclosing their PrEP use would put them at risk of being perceived to be engaging in risky sexual behaviour and stigmatised by people in their community who, due to lack of knowledge about PrEP, associated PrEP use with being HIV positive. Some participants had tried to address this community stigma by using their own PrEP use to educate others about the differences between PrEP and antiretroviral treatment (ART) for HIV.

*They were telling me*,* my friends*,* that taking PrEP is like taking ARVs because I have to take it every day and I will have to go to the clinic to take the pills*,* just like people who are taking ARVs. I said*,* “It’s fine*,* at least I know I am HIV negative; it would have been worse if I had to take pills because I am already sick. PrEP is better than ARVs”.****(24 years old***,*** 9 months on PrEP)***.

However, several participants insisted they were not affected by negative comments about PrEP and instead chose to focus on taking their PrEP to have continued protection from HIV infection.

*There was one person who said PrEP…in fact makes me seem like I have HIV and AIDS*,* since I will take the medication until death*,* just like ARVs. I did not care much since I do not give precedence to any opinion against something that would otherwise help me.****(22 years old***,*** 15 months on PrEP)***.

*I do not care what people say. If I decide to do something and I know it will benefit me*,* I just do it*,* regardless of who says what*,* it’s my life. When I’m sick*,* they will be the first ones to say*,* “why were you not preventing?”*,* so I don’t care.****(25 years old***,*** 7 months on PrEP)***.

## Discussion

Our study highlights young women’s experiences and the multi-level influences on PrEP access and use in a real-world setting. We identified factors at various levels of the Socio-Ecological Model, which influenced PrEP access and use among AGYW in primary care settings, although it is also worth noting that many of the factors are applicable across several levels of the SEM.

At the **individual level**, knowledge and awareness of PrEP were positive influences for PrEP use among participants in our study. The educational materials, counselling, and support provided as part of Project PrEP motivated and equipped AGYW to access PrEP services and continue PrEP use. They also derived a keen sense of self-empowerment from their decision to initiate and continue PrEP. In a previous local study, PrEP persistence (defined as at least 12 months of PrEP use) was linked to formation of positive identities where AGYW found the experience of using PrEP empowering and often became PrEP advocates when interacting with their peers [28]. Participants in our study also demonstrated a high awareness of personal vulnerability to HIV based on their lifestyles (for example, having multiple sexual partners, inconsistent condom use, alcohol and drug use), adding to their motivation to use PrEP. The burden of daily pill taking and its association with being sick or using HIV treatment was a noted barrier to PrEP use. As detailed by participants in this study, this was a challenge that may be overcome by long-acting methods. Although side effects were noted by participants, they did not appear to be a key barrier to PrEP use, as AGYW in this study were able to recognise and manage common side effects and develop adherence strategies to stay on PrEP.

At the **interpersonal level**, the role of partners and families in PrEP uptake and use was a key finding as both a facilitator and barrier of PrEP use. Disclosure of PrEP use to family, partners and friends has been reported in other studies to be a positive influencer of continued PrEP use [7, 29, 30]. However, fear and anxiety about their reactions, together with anticipated stigma and a lack of support may be a barrier [29–31]. This highlights the importance of engaging these key groups in messaging and information sharing about PrEP, in order to address myths and misconceptions and elicit their support for young women’s PrEP journeys. The inconsistent condom use reported by many PrEP users in this study highlights the challenges that young women experience with negotiating condom use, and the need for user-controlled HIV prevention options. Strengthened integration of contraceptive, STI and PrEP services is required to ensure that all young women’s health needs are met, whilst supporting them to navigate condom use. The finding that AGYW wanted to use PrEP in order to “be prepared” for anticipated future vulnerabilities is positive and speaks to the need to integrate counselling on HIV prevention and other SRH needs for young women, even if they may not be immediately required.

At the **health system level**, most participants in this study had had positive experiences with PrEP service delivery and reported satisfaction with accessing PrEP services. The concept of youth-friendly spaces was integral to providing services to AGYW in a safe, non-judgemental environment. AGYW in our study appreciated having designated spaces away from the general population accessing clinic services– who tend to be older– as well as short waiting-times, improved privacy, integrated services and friendly clinic staff. Ninsiima et al. also recommend a welcoming environment, privacy, prompt services and positive provider attitudes as some key elements for providing youth-friendly SRH services [32]. Indeed, other studies have found that healthcare provider attitudes play a critical role in PrEP service delivery and have the potential to shape experiences of health services and impact quality of care and uptake of HIV prevention and other SRH services [12–14]. Hence, training healthcare providers on values clarification to address negative attitudes and biases towards AGYW, would be necessary to promote the provision of youth-friendly PrEP services [13, 14]. Key health-system related barriers to PrEP access included distance from the clinic and non-availability of PrEP. Participants who lived far away from the clinic bemoaned having to spend money on transport to access PrEP services and the opportunity cost of missing part of their school programme to attend follow-up visits. Cassidy et al. underscore the importance of providing convenient services that can be integrated into young women’s often unpredictable daily routines [7]. Considering out of facility services and pick-up points, or extended periods between clinical visits, may assist and would respond to user preferences for PrEP services [33]. The occasional stock-outs of PrEP in some clinics were not only inconvenient; they also contributed to interruptions in PrEP use. At the time of project implementation, oral PrEP was only offered in selected primary health care facilities. Some participants therefore attributed missed doses or periods of interrupted PrEP use to not being able to access PrEP at other nearby clinics, which were not participating in the project.

Experience of stigma was the main **community factor** associated with PrEP access and use. Although AGYW were aware of stigma around PrEP use in their communities, they were mostly undeterred by the negative perceptions. It is likely that AGYW felt sufficiently educated about PrEP and therefore empowered and motivated to take PrEP regardless of common misconceptions that stigmatised users. In addition, social support from partners, family and friends played a role in mitigating stigma and facilitated continued PrEP use. AGYW also highlighted important societal factors such as living in unsafe environments where they were at risk of sexual violence as motivation for PrEP use.

Our results suggest that the factors which influence PrEP access and use are often related and evident across all levels of the SEM. AGYW’s perception of their individual vulnerabilities to HIV cannot be isolated from their complex relationships with partners, family, and friends, nor can it be separated from attitudes in the communities to which they belong. Their experiences of health services are similarly impacted by personal and interpersonal factors as well as by structural factors relating to health system weaknesses. The facilitators highlighted in this study such as comprehensive provision of information through counselling and educational resources and supportive healthcare providers should be leveraged to strengthen the support for AGYW and ensure effective PrEP use in this population.

### Strengths and Limitations

A primary strength of this study was the real-world setting and multi-site approach, although in the analysis, data were not stratified by healthcare setting. Secondly, data analysis followed both an inductive and deductive approach, allowing for depth of insights based on themes in the interview guide but also on patterns emerging from the data. The SEM provided a theoretical lens through which to interpret the results, thereby strengthening our organisation of the findings. However, while our study findings provide valuable insights into the experiences of young women in relation to PrEP service delivery and use in the context of routine primary care services in South Africa, we did not explore experiences of other populations who have been a focus of PrEP programmes, such as older women, sex workers and men who have sex with men. In addition, the qualitative study design limits transferability of the findings to other settings. We also acknowledge the risk for response bias regarding experiences of PrEP service delivery and PrEP use, as AGYW may have associated interviewers with the project, and therefore biased their responses to reflect the project– and PrEP taking– in a positive light. Of note, data collection occurred during the COVID pandemic which may have influenced the findings. In particular, restrictions on movement implemented in South Africa during the pandemic may have influenced participants experiences of healthcare and PrEP access.

## Conclusions

PrEP access and use by AGYW in South Africa are influenced by multiple factors at the individual, interpersonal, health system and community level. Whilst interventions to support individual understanding of prevention needs and effective use of PrEP may assist in improving uptake and use, the role of the healthcare provider and health system cannot be underestimated. The findings from this study may contribute to the development of appropriate service delivery strategies for existing and new PrEP products, to ultimately facilitate informed decision-making and access to HIV prevention among young people in South Africa.

## Data Availability

The datasets generated and analysed during the current study are not publicly available but are available from the corresponding author on reasonable request.

## Abbreviations

PrEP: Pre-exposure prophylaxis
AGYW: Adolescent girls and young women
COREQ: Consolidated Criteria for Reporting Qualitative Studies
WHO: World Health Organisation
SRH: Sexual and Reproductive Health
SEM: Socio-Ecological Model
STI: Sexually Transmitted Infection
IQR: Inter Quartile Range
